# Enabling Rapid pH Equilibration Through Acoustic Microstreaming for Efficient pH Regulation in Microliter-Scale Samples with Screen-Printed Electrodes

**DOI:** 10.3390/bios16080424

**Published:** 2026-08-06

**Authors:** Ziyi Xiao, Ruyi Deng, Xin Liu, Jiahuan Zheng, Kaisong Yuan

**Affiliations:** 1 Bio-Analytical Laboratory, Shantou University Medical College, Shantou 515041, China; 20zyxiao@stu.edu.cn (Z.X.); 20rydeng@stu.edu.cn (R.D.); 25xliu@stu.edu.cn (X.L.); 2 Department of Chemistry, Shantou University Medical College, Shantou 515041, China

**Keywords:** pH regulation, micromixing, acoustic microstreaming, screen-printed electrode

## Abstract

Real-time pH measurement and regulation of microliter-scale liquid samples are critical for microfluidic biochemical applications. However, traditional magnetic stirring for macroscopic systems is not applicable under such microscale conditions. This work develops an ultrasound-assisted screen-printed electrode (SPE) pH sensor integrated with a 3D-printed ultrasonic reflection cell and piezoelectric excitation unit. Ultrasound-induced acoustic streaming accelerates mass transfer inside microliter-scale samples. Upon acid addition, the ultrasonic-assisted system reaches potentiometric equilibrium far more rapidly than the static non-ultrasonic control. Optimized parameters are 30 s ultrasonic duration and 5 Vpp input amplitude. The sensor shows linearity from pH 3.04 to 4.15 with R^2^ = 0.9919. Phenolphthalein visualization directly confirms ultrasound-enhanced molecular diffusion. Its mass transfer efficiency matches traditional magnetic stirring, and stable fast equilibrium is realized in complex cell culture medium. This acoustofluidic micromixing strategy offers a simple integrated route for pH monitoring and the regulation of micro-volume biological samples.

## 1. Introduction

Precise pH measurement and regulation are indispensable for stabilizing biological reaction systems and ensuring valid, repeatable experimental results [1,2,3,4]. Real-time pH detection occupies a vital position in multiple biological assays, including on-chip cell culture [5,6], microbial surveillance [7,8,9], and clinical disease diagnosis [10]. Conventional laboratory pH adjustment is achieved either via direct addition of acids or bases within samples. For samples with sufficient liquid volume, potentiometric pH electrodes enable quantitative digital readouts, while pH indicator reagents support colorimetric pH identification.

With the rapid expansion of microfluidic and lab-on-a-chip technologies, biochemical analytical procedures have been increasingly miniaturized and transferred from traditional benchtop platforms to microfluidic chips [11,12,13,14]. Such microsystems are capable of processing trace and valuable biological samples, offering distinct advantages including compact device size and minimal sample consumption [15,16]. Nevertheless, the above conventional pH regulation and detection methods are no longer applicable to such microfluidic systems. Unlike macroscopic laboratory environments, microfluidic chips feature ultra-small channel volumes, miniature reaction spaces, and extremely low sample consumption, which make traditional bulk pH tuning and large-scale detection tools ineffective [17,18]. In microscale confined environments, subtle pH fluctuations can significantly alter microenvironment homeostasis, interfere with biological reactions, and affect the activity and growth of cultured cells. Cappellesso et al. [19] noted in their review that acidification and lactate in the tumor microenvironment can promote tumor progression by suppressing the anti-tumor responses of innate and adaptive immune cells; the microenvironment pH can be as low as 5.6, and the acidification can also promote the proteolytic activity of fibroblasts and tumor-associated macrophages, thereby remodeling the extracellular matrix. Mikkola et al. [20] further found that minute pH fluctuations in the extracellular environment can significantly influence the cytotoxic effects of ammonia. A low extracellular pH can counteract ammonia-induced cell growth inhibition and genomic instability. This demonstrates, from the perspectives of both immune regulation and cell survival, how minute pH fluctuations alter microenvironmental homeostasis, disrupt biological responses, and influence cellular activity through multiple pathways. Therefore, precise, real-time, and in situ pH control and monitoring are particularly critical in microfluidic chip-based biological analysis and cell culture systems, serving as a fundamental prerequisite for stable and reliable microscale experimental results.

To regulate the pH of small volumes of liquid on the microfluidic chip, many groups have proposed their intelligent designs. David Welch et al. [21] developed a microfluidic system capable of precisely regulating solution pH. The system utilizes an extended-gate ion-sensitive field-effect transistor (ISFET) integrated with a pseudo-reference electrode for real-time pH monitoring within the microfluidic reaction chamber. By controlling the flow rates of two input channels using pulse-width modulated signals applied to on-chip integrated valves, the authors achieved accurate and dynamic pH regulation. As highlighted by Rene Welden et al. [22] for biological and chemical systems, the pH value is a crucial factor, and to bring the system out of equilibrium or to control precisely the surrounding medium are required. In their work, they integrate two light-controllable semiconductor devices into microfluidics: an Al/Si/SiO_2_/Si_3_N_4_ light-addressable potentiometric sensor for pH detection and TiO_2_-based light-addressable electrodes for photoelectrocatalytic pH tuning. All these designs fully demonstrate the necessity and significance of on-chip pH regulation. Notably, continuous injection of exogenous H or OH during pH tuning inevitably alters the ionic composition of sample solutions. In this context, sufficient mixing of the newly introduced solution with the original sample is essential, as accurate pH measurement can only be acquired after the achievement of homogeneous solution mixing.

To accelerate solution mixing and achieve rapid pH equilibrium in microfluidic channels, ultrasound has emerged as an efficient and facile technical approach. As a kind of intensity-controllable mechanical wave that can propagate through media, ultrasound can induce microscale stirring effects in solutions during transmission, enabling rapid homogenization of samples and improving the efficiency of pH sensing [23,24,25,26]. Compared with other rapid mixing strategies based on micropumps or fluid flow rate regulation, ultrasound-based excitation devices feature a much simpler structural configuration, which is highly suitable for integrated and miniaturized microfluidic systems. For instance, Li et al. [27] utilized surface acoustic waves (SAWs) to improve both the sensitivity and response speed of electrochemical detection platforms. Two interdigital transducers were integrated to generate SAWs, which further induced acoustic streaming (AS) within the analyte solution in contact with the sensor. Our group has recently employed ultrasound to generate microstreaming within the solution, thereby accelerating analyte mass transport and improving the sensing performance of liquid crystal droplets [28].

To verify whether the ultrasound propulsion can generate a “micro-mixing” effect during the pH regulation process for microvolume samples, facilitating subsequent pH measurements of solutions at equilibrium, we tailor-made an ultrasonic reflection chamber that was coupled to the pH-sensitive screen-printed electrodes (SPEs). Specifically, a miniature ultrasonic reflection cell was fabricated by 3D printing and integrated with a piezoelectric ceramic ultrasonic driving system to achieve rapid acoustofluidic mixing of microliter-scale solutions. Experimental results show that the ultrasound-assisted system can reach potential equilibrium within approximately 30 s after new solutions are injected, while the counterpart without ultrasonic assistance requires about 300 s, leading to a substantial improvement in detection efficiency. Visualized microflow field simulation experiments further verify that ultrasound can significantly accelerate mass transfer and diffusion of molecules. This method exhibits favorable linearity within the pH range of 3.04–4.15 and maintains rapid responsiveness to variations in hydrogen ion concentration even in complex real samples such as cell/bacteria culture media. Additionally, it should be noted that in this work, we demonstrate that ultrasound actuation can improve the equilibrium attainment efficiency of liquid samples during pH adjustment for small-volume liquid systems. This strategy offers a novel route for enhancing mixing efficiency during on-chip pH regulation. Nevertheless, it should be noted that all current experiments are conducted using a custom-built ultrasonic reflection cell for microvolume samples. When implementing pH tuning inside microfluidic channels, bulk acoustic waves may not yield satisfactory reflection performance. Future studies may adopt surface acoustic waves (SAWs) to achieve superior microscale mixing of liquid solutions [29,30,31].

## 2. Materials and Methods

### 2.1. Chemicals and Instruments

Hydrochloric acid (AR) was purchased from Xilong Scientific (Shantou, China). Sodium hydroxide (S140903-500g) and phenolphthalein (P108715-25g) were purchased from Shanghai Aladdin Biochemical Technology Co., Ltd. (Shanghai, China). Ethanol (95%) was sourced from Xilong Scientific Co., Ltd. DMEM/F-12 medium was purchased from Guangzhou Gubao Biotechnology Co., Ltd. (Guangzhou, China) 2216E liquid medium was purchased from Ruichu Biotechnology Co., Ltd. (Jiangsu, China) The nutrient broth NB medium was purchased from Changde Bickman Biotechnology Co., Ltd. (Changde, China).

The hydrogen ion carrier-modified screen-printed carbon electrodes were purchased from Metrohm (China) Co., Ltd. (Shanghai, China). The E-201-C pH composite electrode was purchased from Beijing United Scientific Instruments Technology Co., Ltd. (Beijing, China). Microfluidic chips and chip triple-port valve connectors were purchased from Wuhan Jianmi Zhikong Technology Co., Ltd. (Wuhan, China). PDMS sheets were obtained from Hangzhou Westru Technology Co., Ltd. (Hangzhou, China). The 3D printer was purchased from Shenzhen Bamboo Lab Technology Co., Ltd. (Shenzhen, China). The sine wave signal generator was obtained from Zhengzhou FeelElec Technology Co., Ltd. (FY6300–30M, Zhengzhou, China). The power amplifier was purchased from Shenzhen Aodewang Electronics Technology Co., Ltd. (Shenzhen, China). Piezoelectric Ceramics were purchased from Zhuhai Jiaming Electronic Technology Co., Ltd. (Zhuhai, China). A dual-channel digital storage oscilloscope was obtained from Shenzhen FNIRSI Technology Co., Ltd. (FNIRSI) (Shenzhen, China). The coaxial optical microscope was obtained from Shenzhen Beiyinhu Technology Co., Ltd. (Shenzhen, China).

To ensure the stability and comparability of the electrochemical signals, all electrochemical detection equipment and associated instruments (including the electrochemical workstation, signal generator, power amplifier, oscilloscope and detection apparatus) used in this study were kept in the laboratory on a long-term basis; the ambient temperature of the laboratory was maintained at a relatively stable level by air conditioning. The ultrasonic-assisted group and the control group were tested in parallel under exactly the same environmental conditions; all test components (including SPEs, reflection chambers and solutions) were kept in the same room-temperature environment and maintained at the same temperature as the ambient temperature.

### 2.2. Construction and Calibration of the Ultrasound-Assisted Detection Device

#### 2.2.1. Fabrication of the Ultrasonic Reflection Chamber

The ultrasonic reflection chamber was fabricated using 3D printing technology, with the specific modeling and processing steps as follows: First, a basic rectangular prism structure was designed using 3D modeling software, with dimensions set at 25 mm × 24 mm × 1 mm. Subsequently, a cylindrical cavity with a diameter of 10 mm was hollowed out at the center of the prism. At the rightmost point of the horizontal diameter of this cylinder, a semicircular structure with a radius of 1 mm was constructed, and the overlapping portion with the cylinder was hollowed out to form a side sampling channel, facilitating the precise addition of sample solutions. This completed the modeling and 3D printing of the ultrasonic reflection chamber.

After printing, the ultrasonic reflection chamber was tightly adhered to the surface of a microscope slide (specifications: 2.5 cm × 7.5 cm × 1.16 mm), ensuring no gaps between the reflection chamber and the slide and maintaining a proper seal to prevent sample solution leakage. Ultrasound coupling agent was evenly applied to the underside of the microscope slide, which was then firmly attached to a piezoelectric ceramic plate (specification: cylindrical, 50 mm diameter, 4 mm thickness) to ensure effective transmission of mechanical vibrations.

#### 2.2.2. Assembly of the Ultrasonic Driving System

The ultrasonic driving system consisted of a sine wave signal generator, a power amplifier, and a piezoelectric ceramic plate. The sine wave signal generator was used to output electrical signals with preset ultrasonic frequency and amplitude, which were subsequently amplified by the power amplifier and transmitted to the piezoelectric ceramic. In particular, polystyrene (PS) microspheres were selected as tracer particles to visualize the fluid motion behavior under the influence of ultrasound. A signal generator was used to perform a frequency sweep ranging from 0 to 600 kHz in 10 kHz increments, and the microspheres’ response to the ultrasonic field was observed. The results showed that the microspheres exhibited slight movement at 510 kHz and 530 kHz, with the most pronounced response occurring at 540 kHz; no significant changes were observed at other frequencies. Consequently, 540 kHz was selected as the operating frequency for subsequent experiments. With regard to amplitude optimization, we investigated the effect of the signal generator’s output amplitude, within the range of 0–10 Vpp, on the potential response. The results indicated that when the set amplitude exceeded 6 Vpp, the actual output amplitudegain after amplification by the power amplifier tended to saturate; simultaneously, the power amplifier generated significant heat, which was detrimental to the stable operation of the system. Consequently, we further investigated five levels—1, 2, 3, 4 and 5 Vpp—within the 0–5 Vpp range. The experimental results show that when the amplitude exceeds 3 Vpp, the response signal is significantly enhanced; at 5 Vpp, the response tends towards saturation; and further increasing the amplitude does not improve the mixing efficiency but may, on the contrary, introduce bubble interference. The entire driving system enabled a constant ultrasonic frequency and continuously adjustable amplitude, allowing precise control of ultrasonic intensity and enhancing the acoustic–fluidic micromixing effect within the system.

#### 2.2.3. Commissioning of the Entire Assembly

The electrode adapter was horizontally inverted and secured onto a high-precision lifting platform to ensure that the electrode detection surface remained horizontal after installation. The integrated ultrasonic components (including the ultrasonic reflection chamber, microscope slide, and piezoelectric ceramic plate) were then secured onto an XYZ three-dimensional precision displacement stage (with a Z-axis fine adjustment range of 10 mm). The power amplifier and signal generator were connected after debugging. By adjusting the height of the lifting platform, the distance between the lower surface of the electrode adapter and the surface of the displacement platform was maintained at approximately 5 mm, ensuring precise alignment between the electrode and the reflection chamber.

### 2.3. Investigation of Sample Solution Motion Behavior and Experimental Conditions

In the experiment, the hydrochloric acid sample solutions were first prepared as follows:

(1) Hydrochloric acid solution I: Take concentrated hydrochloric acid and dilute it 6-fold.
(2) Hydrochloric acid solution II: Take hydrochloric acid solution I and dilute it 20-fold.
(3) Hydrochloric acid solution III: Take hydrochloric acid solution II and dilute it 2-fold.
(4) Hydrochloric acid solution IV: Take hydrochloric acid solution III and dilute it 5-fold.
(5) Hydrochloric acid solution V: Take hydrochloric acid solution IV and dilute it 2-fold.
(6) Hydrochloric acid solution VI: Take hydrochloric acid solution V and dilute it 5-fold.
(7) Hydrochloric acid solution VII: Take hydrochloric acid solution VI and dilute it 2-fold.
(8) Hydrochloric acid solution VIII: Take hydrochloric acid solution VII and dilute it 5-fold.

The screen-printed electrodes used in this study were mounted on ceramic substrates measuring 3.4 cm × 1.0 cm × 0.05 cm. The working electrode was a carbon/hydrogen ion carrier-modified electrode, the counter electrode was a carbon electrode, and the reference electrode was a silver electrode. The working electrode had a diameter of 4 mm and a geometric area of 0.11 cm^2^. The corresponding SEM morphological images, energy-dispersive X-ray spectroscopy (EDS) scan maps, and area scan results are shown in Figure S1 and Table S1 of the Supplementary Materials.

A new screen-printed carbon electrode (SPCE) modified with hydrogen ion carriers was taken, and its test surface was oriented downward and precisely fixed onto the electrode adapter. The electrochemical workstation CHI660 series (CH Instruments Inc.) (Shanghai, China) was started, and the circuit was constructed according to standard electrode connection specifications: the red clip wire was connected to the auxiliary electrode, the green clip wire to the working electrode, and the white clip wire to the reference electrode.

In this experiment, the open circuit potential-time (OCPT) method was employed for electrochemical signal detection. The detection parameters were set using the CHI workstation as follows: High E = 0.3 V, Low E = −0.3 V, Run Time = 600 s, and Sample Interval = 0.1 s.

The detection procedure was as follows: 100 μL of deionized water was precisely transferred into the ultrasonic reflection cell. By finely adjusting the Z-axis displacement of the XYZ three-dimensional precision displacement stage, the electrode test surface was brought into gapless contact with the solution, ensuring effective ultrasonic propagation and electrochemical detection. The detection program was initiated, and blank electrochemical signals of 100 μL of deionized water were collected for the first 200 s. At 200 s of detection, 2.5 μL of hydrochloric acid was precisely added to the ultrasonic reflection cell, and signal acquisition continued for the remaining 400 s. The potential variation curve over time was recorded throughout the entire process.

### 2.4. Investigation of the Influence of Different Experimental Conditions

#### 2.4.1. Influence of Ultrasonic Time

The timing of ultrasound activation was synchronized with the addition of the hydrochloric acid solution. To investigate the influence of ultrasonic time on the electrochemical detection signal of hydrochloric acid, hydrochloric acid solution I was selected as the detection object. Under otherwise identical experimental conditions (electrochemical parameters, solution addition volume, electrode contact state, etc.), ultrasonic times of 5 s, 10 s, 20 s, 30 s, and 40 s were respectively set, and the open-circuit potential signals under different ultrasonic durations were collected and recorded.

#### 2.4.2. Influence of Signal Generator Amplitude

The output amplitude of the signal generator directly determines the ultrasonic intensity. An oscilloscope was used to accurately characterize and record the actual output after amplification by a factor of 10 through the power amplifier for original signal generator outputs of 1 Vpp, 2 Vpp, 3 Vpp, 4 Vpp, and 5 Vpp. Using hydrochloric acid solution I as the detection object, the different amplified signals were respectively applied, and detection was completed under identical electrochemical parameter conditions.

#### 2.4.3. Investigation of Linear Relationship for Different Hydrochloric Acid Concentrations

Six gradient concentrations of hydrochloric acid solutions III, IV, V, VI, VII, VIII were selected as detection objects. Three parallel experiments were conducted for each concentration group under constant parameters. Precisely 100 μL of each test hydrochloric acid solution at each concentration was transferred, and electrical signals were continuously collected for 300 s, with the potential variation curve over time recorded throughout. Simultaneously, an E-201-C type combination pH electrode was used to measure the pH of the three hydrochloric acid solutions three times each. The result of each measurement was recorded, and the mean pH for each group was calculated.

The data processing method was as follows: The last 100 voltage signals (sampling interval of 0.1 s, corresponding to voltage data from 290 s to 300 s of detection time) from the three parallel datasets for each test solution were selected. Their mean values were calculated, respectively, and the relative standard deviation (RSD) for each set of parallel data was also calculated to evaluate the repeatability of the detection method. Linear regression analysis was performed using the mean pH of the hydrochloric acid solutions as the independent variable (x) and the corresponding mean voltage values as the dependent variable (y). A linear regression equation was constructed, and the coefficient of determination (R^2^) was calculated to investigate the linear relationship of this detection method within the target concentration range, providing a basis for the quantitative detection of hydrochloric acid.

### 2.5. Conventional pH-Combination Electrode Detection

A Leici combination pH electrode was selected for the detection experiment. First, electrode pretreatment and circuit connection were performed: The lead wire of the Leici pH electrode was cut open, and the wire serving as the reference electrode was connected to the white clip wire of the electrochemical workstation, while the wire serving as the working electrode was connected to the green clip wire. After connection, the electrode detection surface was repeatedly rinsed with ultrapure water to remove surface impurities, and then the surface moisture of the electrode was blotted dry with clean filter paper to avoid residual moisture affecting detection accuracy.

The prepared pH electrode was precisely fixed, ensuring the electrode detection end was vertically immersed in a detection cell containing 50 mL of ultrapure water. The detection program was initiated. After stably collecting blank potential signals for 200 s, precisely 1.25 mL of hydrochloric acid solution II was added to the detection cell. Simultaneously, a magnetic stirrer was started, the rotation speed was adjusted to 1800 rpm, stirring was stopped after 30 s, and the potential change over time was recorded throughout the process. To investigate the influence of magnetic stirring on the detection results, parallel measurements were performed under the same experimental conditions without magnetic stirring, and the relevant detection data were recorded.

### 2.6. Microfluidic Field Visualization Simulation Experiment

To visualize the diffusion behavior of hydrochloric acid solution within the ultrasonic reflection cell, phenolphthalein indicator solution was used as a visualization indicator, leveraging its property of turning red upon contact with alkali to track the solution diffusion process.

Precisely 100 μL of 0.1 mol/L colorless sodium hydroxide solution was injected into the reflection cell, followed by the precise addition of 1 μL of 0.1% phenolphthalein indicator solution. Ultrasound with a frequency of 540 kHz and an actual amplitude of 41.25 Vpp was applied via the signal generator. A coaxial light microscopy system continuously captured the diffusion process of phenolphthalein before and after ultrasound activation. Simultaneously, a blank control group was established, where the free diffusion process of the phenolphthalein indicator was recorded without applying ultrasound.

### 2.7. Analysis of Real Samples

To evaluate the reliability of the established method in actual complex matrices, three different types of culture media were selected to replace deionized water, simulating hydrochloric acid detection scenarios in complex matrices containing hydrochloric acid, in order to verify the system’s feasibility for detection in acidic microenvironments.

DMEM cell culture medium, 2216E liquid medium for the cultivation of marine bacteria, and nutrient broth (NB) medium for microbial enrichment testing were selected as representative real-world samples. A precise 100 μL of the corresponding culture medium was dispensed into the reflection cell. The detection program was initiated; after a steady 200 s of blank signal acquisition, a precise 5 μL of hydrochloric acid solution I was dispensed into the reflection cell. Two experimental conditions were set up—with and without ultrasonication—and signal acquisition was completed for the remaining 400 s.

## 3. Results

### 3.1. Construction and Debugging of Ultrasound-Assisted SPE pH Sensor

pH serves as a pivotal indicator of physiological and biochemical status, and its precise determination is especially critical for microvolume biological samples such as intracellular or extracellular fluid [31]. Due to the limited volume, such samples cannot be analyzed using conventional pH measurement devices. Screen-printed electrodes (SPEs) are compact sensing electrodes widely employed in the electrochemical research area, making them highly suitable for pH detection in microvolume samples [32]. In this work, a 3D-printed reflection cell was designed for holding microvolume samples, enabling its integration with SPEs for pH measurement. In contrast to traditional pH detection systems, which require large sample consumption, the proposed device meets the analytical demand in sample-limited scenarios and effectively addresses the technical challenge of detecting microvolume samples.

The uniform distribution of hydrogen ions (H^+^), which requires sufficient mixing to avoid detection deviations caused by localized inhomogeneities in the H^+^ concentration, is essential for accurate pH measurement. Magnetic stirrers equipped with matching magnetic stir bars are commonly employed in conventional pH measurement to mechanically agitate the solution, guaranteeing accurate results [33]. However, owing to the constraints imposed by the microscale sample volume and the design of our device, ultrasonic mixing was adopted instead of conventional mechanical stirring. The implementation is detailed as follows: A sine-wave signal generator, a power amplifier, and a piezoelectric ceramic plate are integrated and assembled. The signal generator outputs an electrical signal with a specific frequency and amplitude, which is then amplified by the power amplifier and transmitted to the piezoelectric ceramic plate. The piezoelectric ceramic plate converts the electrical signal into mechanical vibration, thereby generating ultrasonic waves with stable frequency and controllable amplitude. By precisely adjusting the output parameters of the signal generator and the amplification factor of the power amplifier, the ultrasonic intensity can be accurately controlled. The cavitation effect and acoustic streaming induced by ultrasound [34] accelerate the mixing process of the microvolume sample solution, enabling H^+^ to rapidly achieve a uniform distribution throughout the solution system, and ultimately allowing rapid and accurate detection of the sample pH.

**Figure 1:**
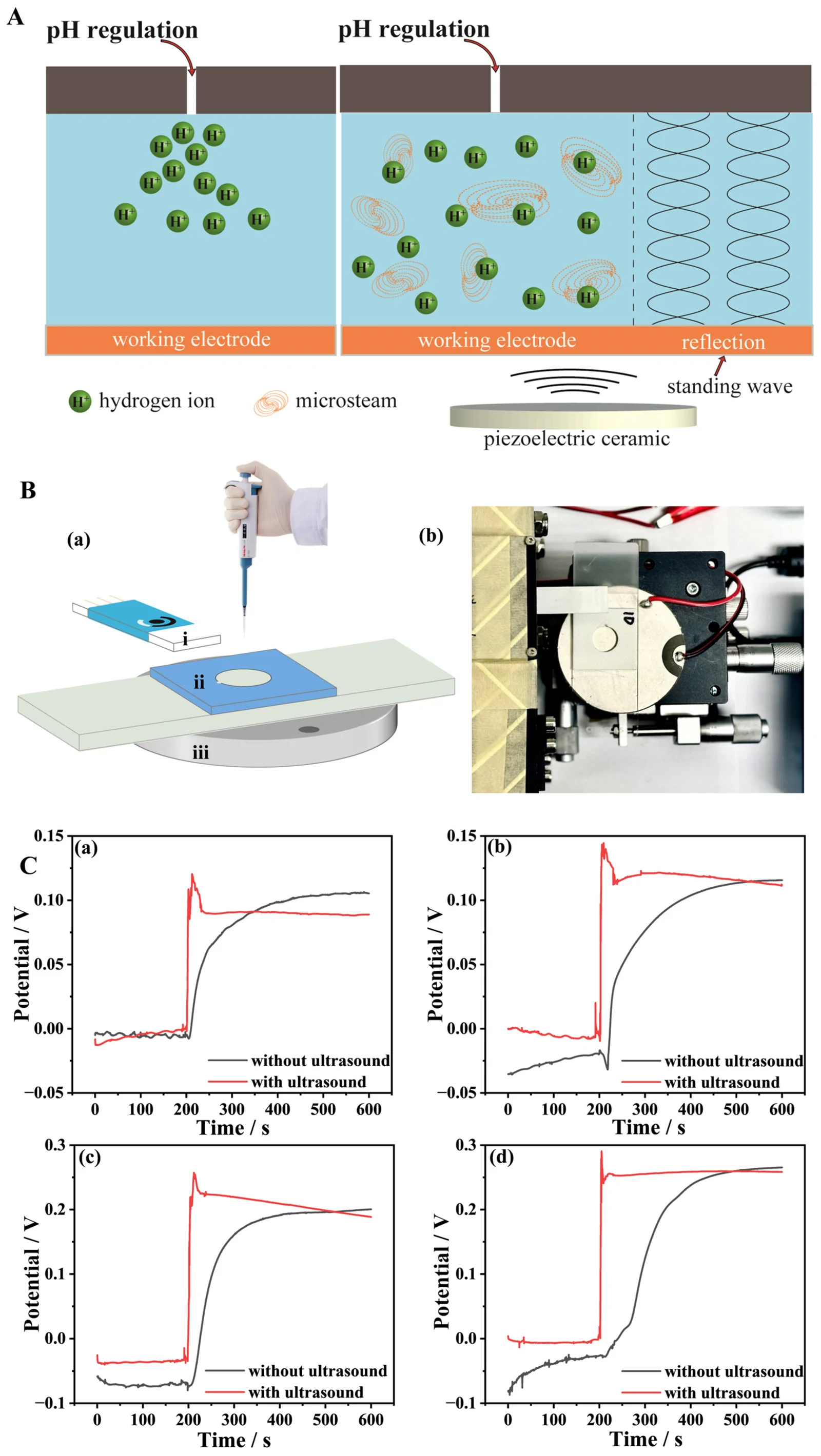
(**A**) pH measurement strategy of ultrasound-assisted SPE pH sensor, and (**B**) an image of the device core assembly where (**a**) is the render image ((i) screen-printed electrode (SPE), (ii) reflection cell, and (iii) piezoelectric ceramic plate) and (**b**) is the real image, and (**C**) is the potential curves of hydrochloric acid at different concentrations with or without ultrasound ((**a**) hydrochloric acid solution V, (**b**) hydrochloric acid solution IV, (**c**) hydrochloric acid solution II, and (**d**) hydrochloric acid solution I).

Figure 1A illustrates the strategy schematic of the integrated device. The reflection cell, which holds the sample solution and reflects ultrasound to form standing waves, is a cylindrical structure 10 mm in diameter and 1 mm in height. A semicircular structure 1 mm in radius is attached to the side of the reflection cell as a sample loading well to enable precise sample introduction. The piezoelectric ceramic plate is closely attached to the bottom of the reflection cell to efficiently receive ultrasonic vibration and achieve sample mixing. Figure 1B(a) shows a rendered image of the core assembly of the device, and (b) presents a photograph of the actual core assembly.

After the device was assembled, a series of standard hydrochloric acid solutions (I, II, IV, V) were used for device debugging. As shown in Figure 1C, both the non-ultrasound system and the ultrasound-assisted system responded to the potential values of hydrochloric acid solutions at different concentrations. The higher voltage was shown in the higher H^+^ concentration solution, which indicates that the pH sensing device composed of SPEs combined with the ultra-small volume 3D-printed reflection cell can effectively determine the pH of solutions. Notably, the ultrasound-assisted system reached equilibrium much faster during measurement, significantly improving the detection speed. When the voltage readings measured by the 200 s system stabilized, hydrochloric acid solutions of varying concentrations were added; the ultrasound-assisted system stabilized again within approximately 30 s, whereas the non-ultrasound system took about 300 s to reach an equilibrium.

### 3.2. Optimization of Experimental Parameters for Ultrasound-Assisted SPE pH Sensor

**Figure 2:**
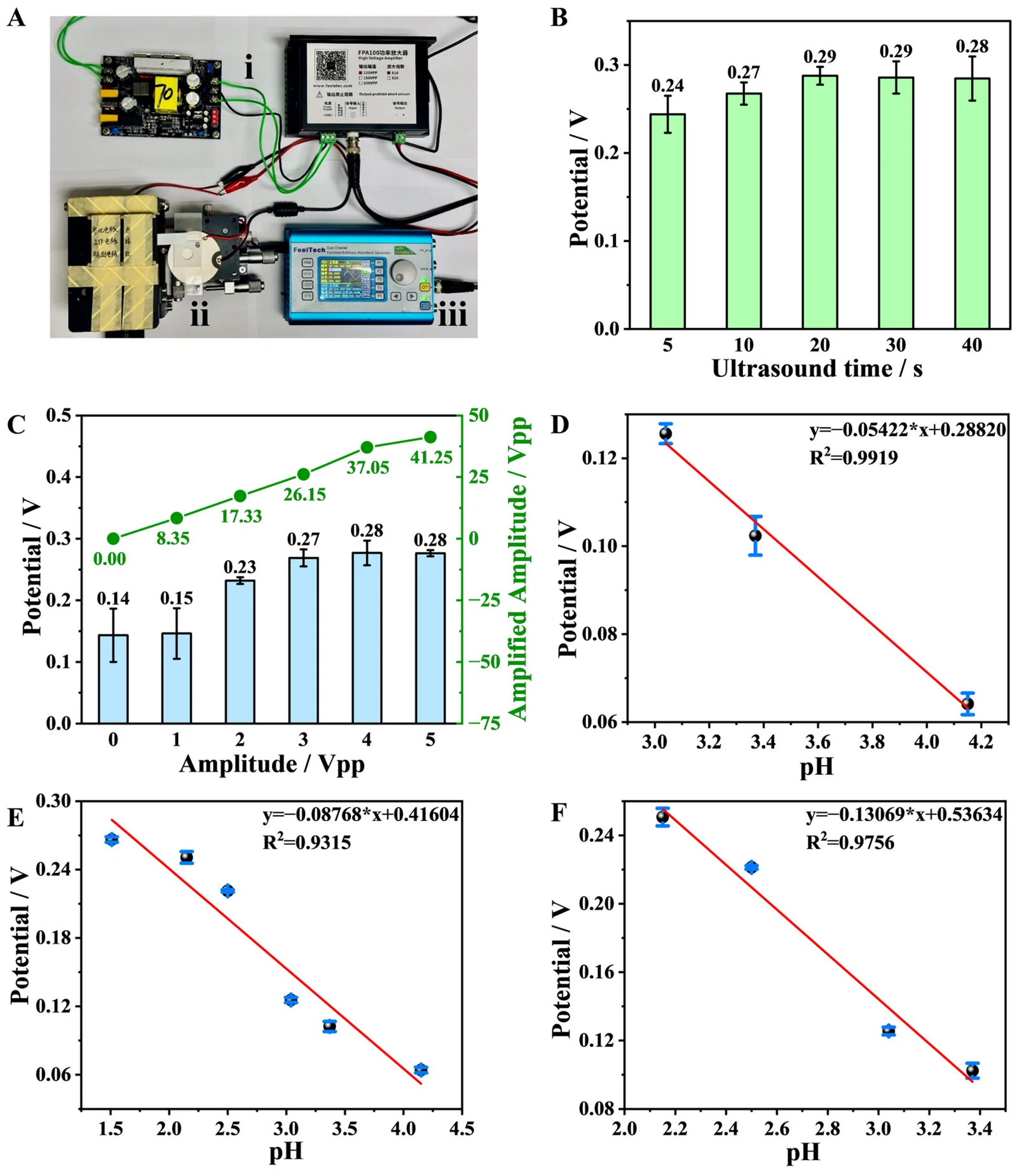
Parameter optimization of the ultrasound-assisted SPE pH sensor: (**A**) Photograph of the complete device, (i) is power amplifier, (ii) is ultrasonic reflection chamber, (iii) is sine wave generator, (**B**) the potential response of hydrochloric acid solution I with different ultrasonic treatment time, (**C**) the potential response of hydrochloric acid solution I under different ultrasonic amplitude, (**D**) the linearity relationship for hydrochloric acid solutions with varying pH values, including three calibration points, (**E**) six calibration points, and (**F**) four calibration points.

Since the potential reaches equilibrium within 30 s after the sample addition at 200 s, the potential value at 230 s was used to evaluate the measurement conditions in subsequent experiments. After assembly and preliminary debugging (Figure 2A), the detection conditions were optimized in terms of ultrasonic time, ultrasonic amplitude, and the linear range of the method.

#### 3.2.1. Ultrasonic Time

Ultrasound accelerates homogenization via the microconvection effect, enabling the system to rapidly reach a uniform and stable state. Nevertheless, the ultrasonic duration exerts a notable impact on both detection efficiency and system stability. An insufficient ultrasonic time fails to achieve thorough mixing, whereas an overly long duration may give rise to problems such as system temperature rise, variations in dissolved gases, and unnecessary energy consumption [35].

In this experiment, hydrochloric acid solution I was primarily employed to investigate the influence of ultrasonic duration on the open-circuit potential. As displayed in Figure 2B, the potential increased gradually with ultrasonic time when the duration was below 20 s, indicating that ultrasound continuously facilitated solution homogenization. Upon extending the ultrasonic treatment to 20 s, the potential recorded at 30 s and 40 s remained nearly identical to that at 20 s, demonstrating that the solution had reached a sufficiently uniform state. Further prolonging the ultrasonic time provided no additional improvement in mixing efficiency but might compromise the stability of potential detection owing to interfacial disturbance, temperature fluctuation, or bubble interference.

It should be noted that for strong electrolyte acids, even at moderate concentrations, the solution pH is predominantly governed by the concentration of H^+^. Short-term ultrasonication does not significantly change the bulk pH because the concentration of H^+^ introduced by acoustic effects is negligible compared with the original hydrogen ion concentration in the solution. Therefore, the stabilization of the potential after 20 s does not arise from changes in pH but directly reflects that the sample has reached a homogeneous state. Further extending the ultrasonic duration yields no further improvement in mixing uniformity. Taking the above results into account and considering that lower concentrations of hydrochloric acid solution require a longer equilibration time, a finite ultrasound duration of 30 s was adopted for this experiment.

#### 3.2.2. Ultrasonic Amplitude

Ultrasonic amplitude is another key parameter affecting micro-mixing effect. An amplitude that is too low will fail to effectively stimulate microstreaming in the solution, while an excessively high amplitude may lead to the formation of bubbles and other undesirable factors that compromise electrode stability. It is therefore crucial to determine a suitable amplitude. Figure 2C illustrates the effect of ultrasonic amplitude on the detection of hydrochloric acid solution I. The x-axis represents the input ultrasonic amplitude, whereas the y-axis on the right shows the actual output amplitude after amplification by the power amplifier. As shown in the results in the figure, when the input amplitude was increased from 0 Vpp to 1 Vpp (actual output 8.35 Vpp), the measured potential rose only slightly from 0.14 V to 0.15 V, indicating that the ultrasonication had a limited effect on promoting the homogenization of the solution within this amplitude range. Upon further increasing the input amplitude to 2 Vpp and 3 Vpp, the actual output reached 17.33 Vpp and 26.15 Vpp, respectively, whilst the measured potential values correspondingly rose to 0.23 V and 0.27 V. This significant enhancement in response indicates that the ultrasound has effectively accelerated the uniform distribution of hydrogen ions within the solution, enabling the electrode surface to come into full contact with the ions under test. When the input amplitude was increased to 4 Vpp and 5 Vpp, the actual outputs were 37.05 Vpp and 41.25 Vpp, respectively, whilst the measured potential stabilized at approximately 0.28 V. This indicates that the ultrasonic effect had approached saturation under these conditions, and that further increases in amplitude would have only a limited effect on improving mixing efficiency. As the fluctuations in the potential value were smaller at an amplitude of 5 Vpp, 5 Vpp was selected as the operating amplitude for subsequent experiments to ensure a stable and sufficient ultrasonic effect.

#### 3.2.3. Linear Relationship

Figure 2D–F illustrates the linear relationship of the proposed method within a certain range. Six hydrochloric acid solutions of different concentrations were selected, namely concentrated hydrochloric acid solutions III, IV, V, VI, VII, VIII. The pH values of the six solutions were determined using a conventional pH composite electrode and found to be 1.51, 2.15, 2.50, 3.04, 3.37, and 4.15, respectively. The results indicate that within the pH range of 3.04–4.15, this method exhibits a good linear relationship, with an R^2^ of 0.9919. A satisfactory linear relationship was also observed in the pH range of 2.15–3.37, with an R^2^ of 0.9756. However, when the highest concentration point (hydrochloric acid solution III, pH approximately 1.51) was included in the overall six-point fit, the linear R^2^ decreased to approximately 0.9315, indicating that the sensor exhibits a response deviation under strongly acidic conditions (pH < 2.0). To evaluate the influence of electrode surface morphology on pH detection, we have provided SEM images and EDS elemental mapping results of the commercial SPE in Figure S1 of the Supplementary Materials. The SEM images reveal a uniform surface morphology with no visible contamination, ensuring stable potential response throughout the experiment. Furthermore, the carbon electrode surface was pre-modified with immobilized hydrogen ion carriers, enabling direct pH detection and ensuring consistency between batches and experimental reproducibility. Compared with platinum electrodes, carbon materials exhibit superior compatibility with ion-carrier polymer membranes and are commonly used in this type of solid-state potentiometric pH sensor. The novelty of this study is the validation of the ultrasonic microfluidic mixing strategy, rather than electrode material optimization; therefore, commercial carbon electrodes are sufficient to support our conclusions.

Moreover, we consider this deviation stems primarily from three factors: protonation saturation of the ion carrier at high H^+^ concentrations, changes in the protonation state of functional groups on the carbon electrode surface affecting the interfacial potential, and potential drift at the reference electrode’s liquid junction under high ionic strength. Conventional glass pH electrodes also exhibit similar deviations under strongly acidic conditions. Therefore, we have defined the sensor’s effective linear range as pH 3.04–4.15 (R^2^ > 0.99) to objectively evaluate its performance.

### 3.3. Ultrasound-Assisted SPE pH Sensor Improves the Efficiency of pH Measurement

**Figure 3:**
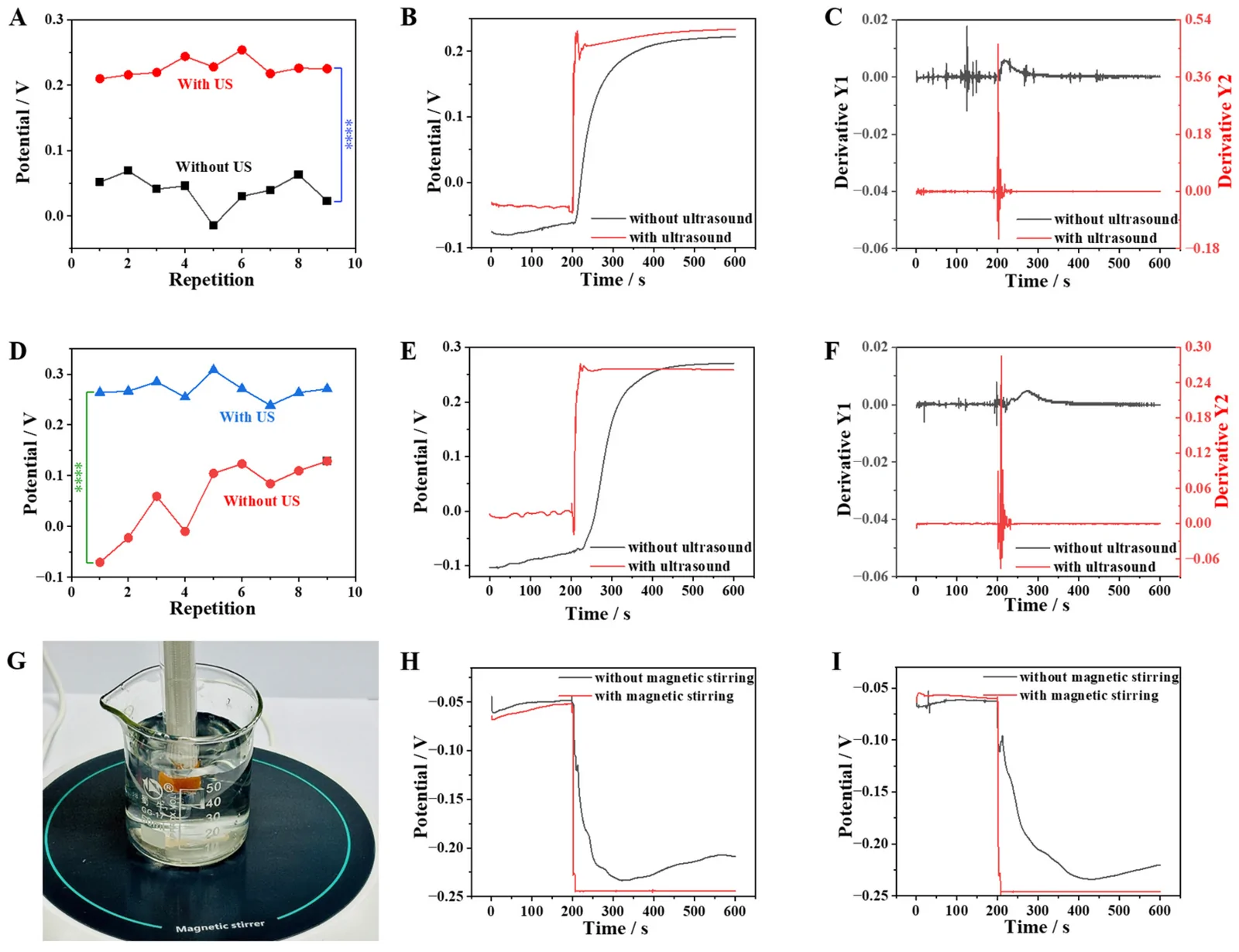
Ultrasound-assisted SPE pH sensor for improving pH measurement efficiency: (**A**,**D**) are nine repeated potentials of hydrochloric acid solution II and hydrochloric acid solution I, respectively, at 230 s. (**B**,**E**) are typical potential curves of hydrochloric acid solution II and hydrochloric acid solution I, respectively. (**C**,**F**) are instantaneous slope curves of (**B**,**E**,**G**), which is the photograph of the conventional mechanical stirring system with large electrodes. (**H**,**I**) are potential curves measured by the system shown in (**G**). **** *p* < 0.0001.

Based on the results shown in Figure 1C, we used hydrochloric acid solutions of varying concentrations to further verify whether ultrasound could improve the efficiency of pH measurement by SPEs. Figure 3A,D present the potentials of two concentrations of hydrochloric acid solutions at 230 s over nine replicate measurements. It can be seen that the potentials at 230 s in the ultrasound-assisted system were significantly higher than those in the non-ultrasound system (*p* < 0.0001), indicating that the ultrasound-assisted system accelerated the diffusion of H^+^ and enabled the system to respond more rapidly to high concentrations of H^+^.

Figure 3B,E are representative curves of the entire measurement process for hydrochloric acid solutions I and II, respectively. As shown, the potential of both concentrations of solutions in the ultrasound-assisted system increased rapidly after 200 s and reached equilibrium (at 200 s, when hydrochloric acid was added and ultrasound was initiated). In contrast, in the non-ultrasound system, the onset of potential increase occurred later than in the ultrasound system, and the potential reached the measurement plateau at a much slower rate, requiring significantly longer time than the ultrasound system. These results further demonstrated that the ultrasound-assisted system improved the efficiency of pH detection.

To quantitatively assess the enhancement effect of ultrasound on detection efficiency, we differentiated the representative potential–time curves using the “Differentiate” tool in Origin software, yielding the instantaneous slope curves. The resulting derivative curves are presented in Figure 3C,F. Both panels feature dual vertical axes: the left axis shows the slope data for the without-ultrasound group (Derivative Y1), and the right axis displays the slope data for the ultrasound group (Derivative Y2). Focusing on the time point of hydrochloric acid addition (200 s), upon introduction of hydrochloric acid solution, the ultrasound-assisted system immediately exhibited a sharp and pronounced peak in the slope, followed by a rapid decline. This observation indicates that ultrasound accelerates the rapid diffusion of H^+^, thereby enabling a swift and homogeneous distribution of hydrogen ions. In contrast, the instantaneous slope peak of the without-ultrasound system was considerably lower, and the curve displayed only minor fluctuations, suggesting that the hydrogen ion concentration in the system continued to fluctuate and required a longer period to achieve a stable distribution.

To compare the effects of the ultrasound-assisted system and the conventional mechanical stirring system on improving pH detection efficiency, and to further verify that the ultrasound-assisted system enhanced the mass transfer process of the solution, a conventional large electrode was also used to detect hydrochloric acid solution II. The photograph of the conventional mechanical stirring system and the results are shown in Figure 3G–I. As illustrated, the potential of the conventional large electrode reached equilibrium rapidly after stirring. Its response trend was essentially consistent with that of the present ultrasound-assisted detection device, indicating that the device has a mass transfer enhancement effect comparable to conventional mechanical stirring.

### 3.4. Visualization Simulation for the Fluid in the Chamber

**Figure 4:**
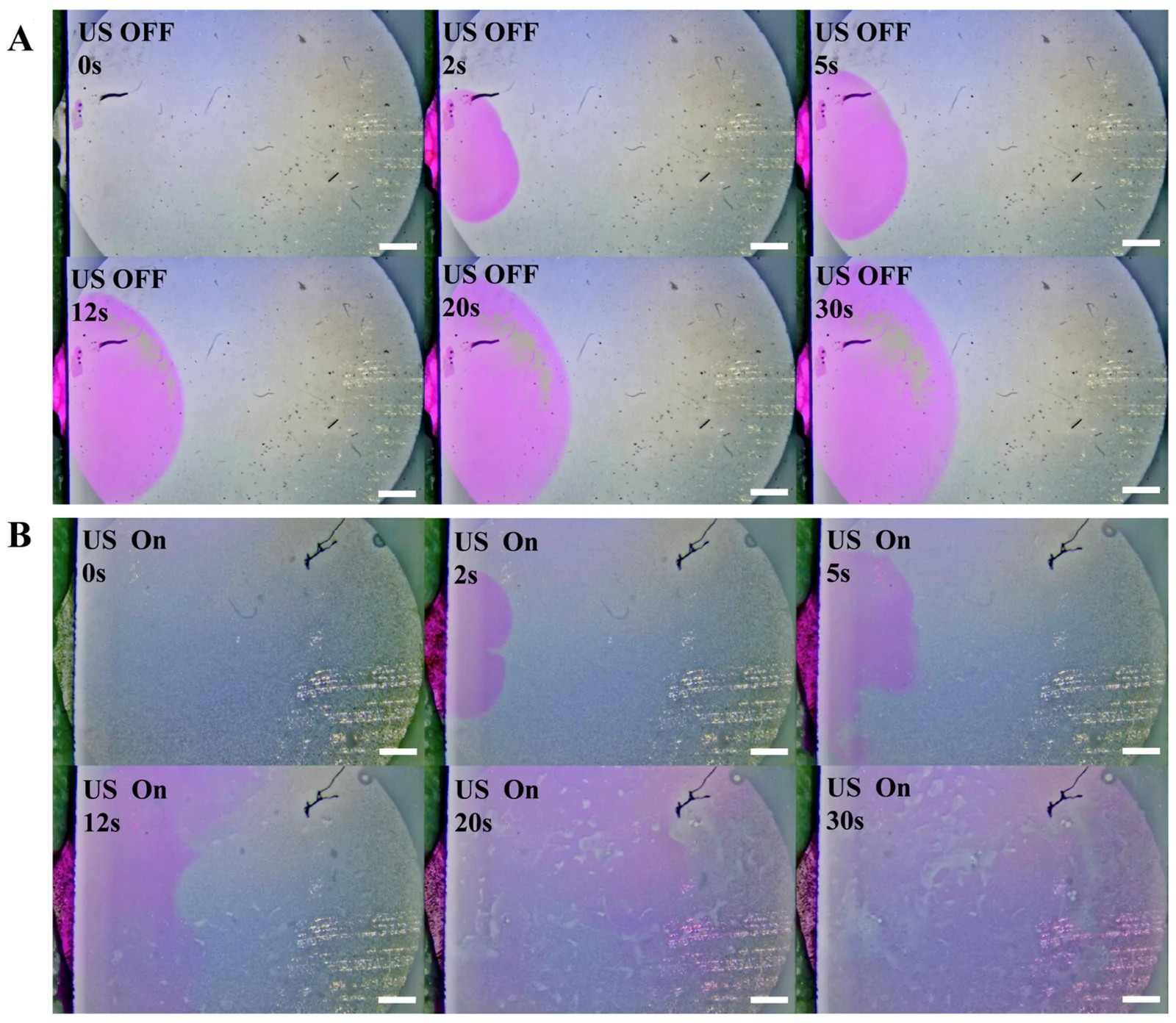
Visualization simulation for the fluid in the chamber: (**A**) Phenolphthalein diffusion with ultrasound OFFand (**B**) ultrasound ON (Scale bar = 1000 μm).

To get further insights into the diffusion behavior of hydrochloric acid solution in the ultrasonic reflection cell, simulation experiments are conducted. Phenolphthalein was adopted as a visual tracer for its characteristic of turning red under alkaline conditions. As shown in Figure 4, phenolphthalein would diffuse throughout the whole ultrasonic reflection cell with US OFF (Figure 4A) and US ON (Figure 4B). As can be seen from the figures, the indicator diffusion rate in both the ultrasound-assisted system and the non-ultrasound system was similar within the initial 5 s. This may be attributed to the response lag of ultrasound-enhanced diffusion due to the cavitation equilibrium time [36]. We speculated that spontaneous diffusion and weak acoustic microstreaming dominate the initial stage, leading to a diffusion rate close to that without an ultrasonic field. Mass transfer accelerates remarkably after cavitation and intense convection fully forms. Starting from 12 s, the diffusion rate of the ultrasound system is obviously higher than that without ultrasound. At 20 s, phenolphthalein basically diffused throughout the reflection cell under ultrasonic conditions, while it only achieved limited diffusion in the non-ultrasound system. This result is consistent with that shown in Figure 2B.

Subsequent observation of phenolphthalein diffusion revealed that its diffusion rate became extremely slow without ultrasonic stimulation, making uniform distribution difficult to achieve, which interferes with the accurate detection of hydrogen ion concentration. Figure 4 presents the images of late-stage phenolphthalein diffusion and the specific dimensions of the ultrasonic reflection cell, further verifying the experimental phenomena.

### 3.5. Simulated Detection for Actual Samples

**Figure 5:**
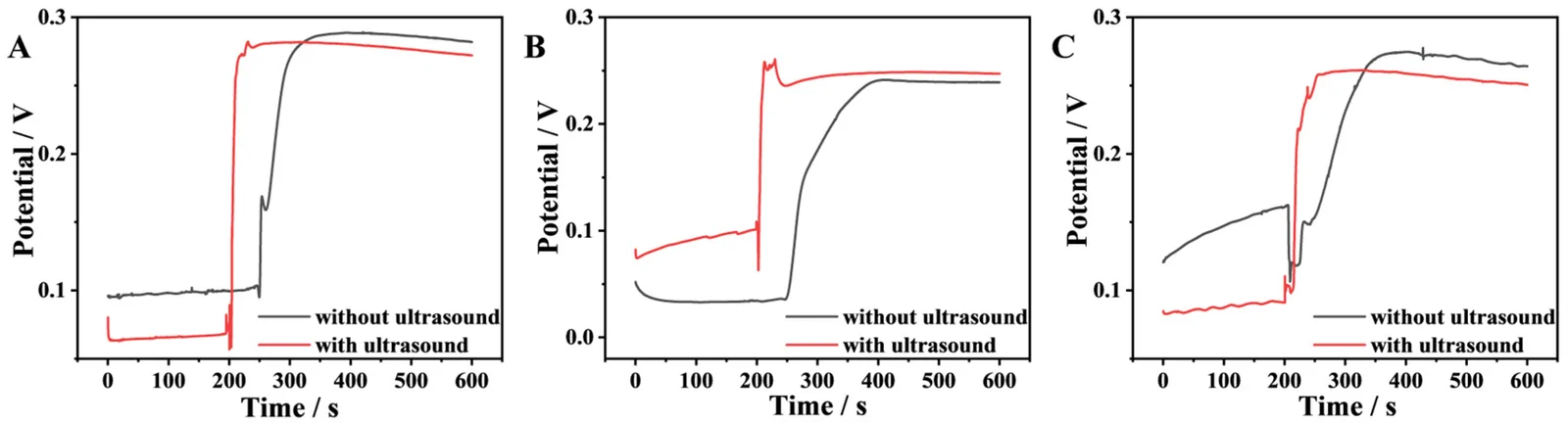
Comparative measurement results with and without the use of ultrasound following the addition of acid to different culture media: (**A**) DMEM cell culture medium, (**B**) nutrient broth (NB) medium, and (**C**) 2216E medium.

A complex matrix environment is a major challenge in actual sample detection, especially for biological specimens. To evaluate the detection feasibility of the ultrasound-assisted SPE pH sensor, DMEM cell culture medium, nutrient broth (NB) medium, and 2216E medium were selected to replace deionized water, and simulated detection experiments for hydrochloric acid solutions were carried out. As shown in Figure 5, in all three culture medium systems, the baseline remained stable prior to the addition of acid, following the addition of hydrochloric acid solution I, a marked response in the system’s potential was observed upon detection of hydrogen ions, and the system tended towards equilibrium after a certain period, indicating that the device can effectively perform pH detection under various complex matrix conditions. A further comparison of the potential change curves under conditions with and without ultrasound reveals that, following the application of ultrasound, the potentials of all three culture medium systems reached equilibrium more rapidly, with response times significantly reduced. This demonstrates that ultrasound plays a significant role in accelerating the uniform distribution of hydrogen ions within complex matrices and enhancing electrode response. These results provide experimental evidence for the realization of rapid and accurate pH detection in future applications involving trace samples of complex matrices or microenvironmental systems.

## 4. Conclusions

Traditional magnetic stirring devices are bulky and unsuitable for microliter-scale liquid samples, rendering them unsuitable for matching SPE-based pH sensing. To tackle this challenge, we design a 3D-printed ultrasonic reflection chamber integrated with an ultrasound generation device (including a piezoelectric ceramic, sine wave signal generator, and power amplifier) to be integrated with SPEs. The ultrasonic waves propagating within the tailor-made chamber excite robust acoustic microstreaming flow fields. This acoustically induced microstreaming dramatically boosts molecular mass transport and homogenizes uneven hydrogen ion distribution across the microliter sample volume, solely cutting down the waiting duration for the whole solution system to reach uniform potentiometric pH equilibrium.

The influence of different experimental parameters, including ultrasonic treatment time (5 s, 10 s, 20 s, 30 s, 40 s) and ultrasonic amplitude (0 Vpp, 1 Vpp, 3 Vpp, 5 Vpp), was systematically studied to acquire the optimal activation conditions. Upon acid injection, the ultrasonic-assisted system attains stable potentiometric equilibrium within only 30 s, while the static control requires about 300 s of self-diffusion to reach uniform concentration distribution. These results also serve as indirect evidence for the enhanced mass transport. In contrast, from the repeatability measurements, we can conclude that the microstreaming generated by ultrasound accelerates the detection system equilibration upon the addition of fresh acidic solutions during pH regulation. Phenolphthalein was utilized to directly visualize the real-time solute diffusion process within the tailor-made chamber, clearly demonstrating that ultrasound drastically accelerates molecular mass transfer in solution. Linear calibration tests revealed that the SPEs-based sensor achieves linear response over the pH range of 3.04–4.15 (R^2^ = 0.9919). Experiments regarding the use of conventional large-volume pH electrodes coupled with magnetic stirrers verified that the proposed ultrasonic mixing strategy achieves comparable mass transfer enhancement efficiency. More importantly, the as-proposed ultrasound-generated microstreaming method could also be used to fasten pH equilibrium in complex cell culture medium matrices, proving its reliability for actual biological micro-sample analysis.

Furthermore, we conducted a comparison of this method with representative microfluidic pH-related systems, demonstrating that existing systems typically rely on passive diffusion or fluid flow to achieve sample homogenization, whereas this work introduces ultrasonic-induced acoustic microfluidics as an active mixing strategy to accelerate the equilibration process. For further details, please refer to Table S2 in the Supplementary Materials. Recent studies have also demonstrated that screen-printing technology can now be extended to flexible substrates for wearable sensing applications [37,38,39]. Liquid-metal stretchable electrodes can be patterned using colloidal self-assembly and micro-transfer techniques, achieving feature sizes as small as 5 μm and strain rates of up to 1200 per cent; ultra-thin, substrate-free liquid-metal electrodes can also be directly applied to human skin for continuous health monitoring [40,41]. In future work, we plan to combine the acoustic-microfluidic mixing strategy validated in this study with flexible electrode platforms, either by designing SPEs in-house or by introducing novel electrode materials to further enhance performance.

## Data Availability

The data presented in this study are available upon request from the corresponding author.

## Supplementary Materials

The following supporting information can be downloaded at https://www.mdpi.com/article/10.3390/bios16080424/s1, References [42,43] are cited in the supplementary materials.
