# Supplementary material for: Enabling Rapid pH Equilibration Through Acoustic Microstreaming for Efficient pH Regulation in Microliter-Scale Samples with Screen-Printed Electrodes
Source: Biosensors (Basel). 2026 Aug 6;16(8):424. doi: 10.3390/bios16080424 (PMC13510799; doi:10.3390/bios16080424)
Supplement: Supplementary file 1 [file biosensors-16-00424-s001.zip › biosensors-4426530-supplementary.pdf]

Enabling Rapid pH Equilibration through Acoustic Microstreaming for Efficient pH Regulation in Microliter-scale Samples with Screen-Printed Electrodes

Ziyi Xiao[1], Ruyi Deng[1], Xin Liu[1], Jiahuan Zheng[2,*] and Kaisong Yuan[1,*]

[1]Bio-Analytical Laboratory, Shantou University Medical College, Shantou 515041, China;

[2]Department of Chemistry, Shantou University Medical College, Shantou 515041, China;

*****Correspondence: jiahuanzheng@stu.edu.cn, ksyuan@stu.edu.cn

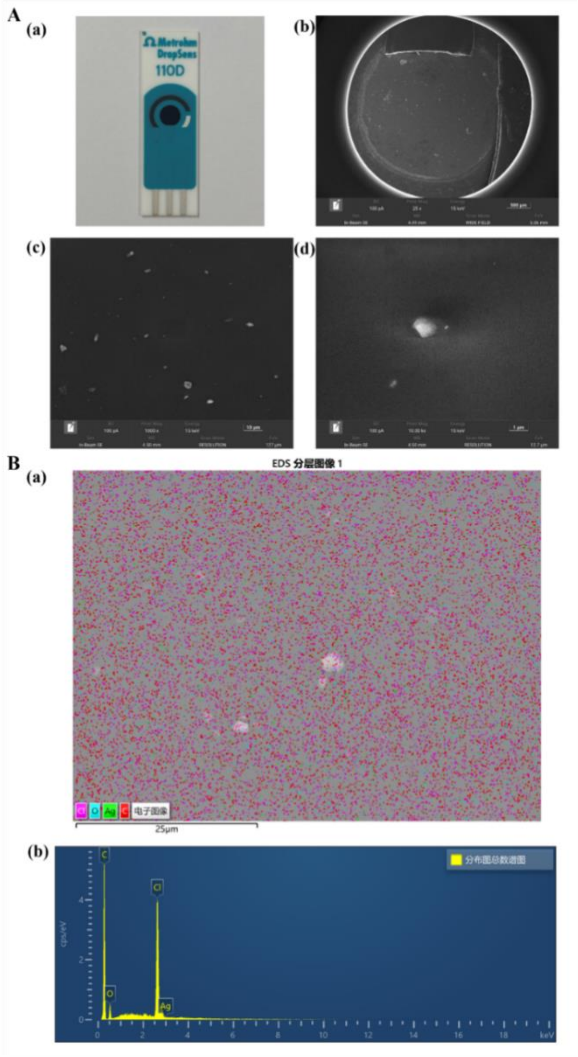

Figure S1 Characterisation of Screen-Printed Carbon Electrodes, **(A)** Photograph of the electrode (a), and SEM images of the working electrode surface at different magnifications (b-d), **(B)** General Map of Element Distribution in the Surface Survey (a), and EDS surface scanning results (b)

Table S1. Results of the EDS surface scanning.

Elements	Line type	Weight percentage	Wt % Sigma	Atomic percentage
**C**	K-Line System	85.98	0.45	92.23
**O**	K-Line System	6.05	0.41	4.87
**Cl**	K-Line System	7.97	0.19	2.89
**Ag**	L-Line System	0.00	0.11	0.00
**Total volume**		100.00		100.00

Table S2. Comparison of the proposed method with representative microfluidic pH-related systems.

System design	Mixing method	Application scenario	Ref
Digital microfluidics with integrated fluorescent pH sensor film	Passive diffusion	Real-time monitoring of extracellular acidification in cancer cells	[1]
Light-addressable potentiometric sensor with light-addressable electrode	Fluid flow, no active mixing	Automated pH control on microfluidic chips	[2]
Paper-based microfluidics with screen-printed electrodes	Capillary-driven wicking and diffusion	Monitoring sweat pH	[3]
SPE with 3D-printed ultrasonic reflection chamber	Ultrasound-induced acoustic microstreaming	Rapid pH equilibration of microlitre samples	This work

References

1. Qiu, W.; Lin, X.; Nagl, S. In Situ Live Monitoring of Extracellular Acidosis near Cancer Cells Using Digital Microfluidics with an Integrated Optical pH Sensor Film. *Analytical Chemistry* **2024**, *96*, 14456-14463.

2. Welden, R.; Das, A.; Krause, S.; Schöning, M. J.; Wagner, P. H.; Wagner, T. Actively Driven Light-Addressable Sensor/Actuator System for Automated pH Control for the Integration in Lab-On-A-Chip (LoC) Platforms. *ACS Sensors* **2024**, *9*, 1533-1544.

3. Fiore, L.; Mazzaracchio, V.; Antinucci, A.; Ferrara, R.; Sciarra, T.; Lista, F.; Shen, A. Q.; Arduini, F. Wearable electrochemical device based on butterfly-like paper-based microfluidics for pH and Na+ monitoring in sweat. *Microchimica Acta* **2024**, *191*, 580.
